# What Is Phenomenology For? Applications in Autism Mental Health and a Pragmatist Criterion of Success

**DOI:** 10.1159/psp/aelag003

**Published:** 2026-08-27

**Authors:** Themistoklis Pantazakos

**Affiliations:** a Honorary Research Associate, Department of Science and Technology Studies, University College London, London, UK; b Department of Psychology, The American College of Greece, Athens, Greece; c Department of Psychology and Educational Sciences, KU Leuven, Leuven, Belgium

**Keywords:** Phenomenology, Autism, Neurodiversity, Mental health, Psychotherapy, Pragmatism, Verification, Epistemic injustice

## Abstract

**Background:**

Phenomenological approaches have gained increasing traction in autism mental health studies, yet what phenomenology specifically contributes, beyond a general orientation toward lived experience, has received little scrutiny. This paper asks two questions: why phenomenology, rather than other ways of attending to experience, is needed in autism mental health research and clinical practice, and what, concretely, it is for. Throughout, it adopts the neurodiversity-affirming stance that autism is not a deficit at the level of the condition, while treating what serves a given person as open, facet by facet, to empirical inquiry.

**Summary:**

The paper makes two contributions. The first is a pragmatist criterion of phenomenological success, according to which an interpretation earns credibility through the predictions and interventions it allows – in the clinical case, its capacity to facilitate outcomes the service user values. However, left unqualified and because it reaches beneath what a person can by themselves articulate, this kind of inquiry risks re-enacting the displacement of autistic voices a neurodiversity-affirming stance opposes. In response, two safeguards are developed to keep interpretation answerable to the person it concerns. The second contribution identifies three sites in autism mental health where phenomenology does work other methods cannot: assessment and formulation, where it surfaces the experiential structure a presenting symptom obscures; evaluation of outcomes, where it supplies the register in which success can be judged at all; and the therapeutic relationship. Illustrations are drawn from a recent qualitative study with autistic psychotherapy clients.

**Key Messages:**

Phenomenology’s contribution to autism mental health is distinctive and non-substitutable, not merely a general orientation toward lived experience. A pragmatist criterion judges a phenomenological interpretation by its capacity to facilitate outcomes the service user values. Phenomenology does uniquely important work at three sites: assessment and formulation, evaluation of outcomes, and the therapeutic relationship.

## Introduction

The neurodiversity movement, which centrally claims that certain neurodevelopmental conditions represent value-neutral deviations from the norm rather than deficits, has taken centre stage in autism studies over the last 2 decades. What began as a niche grassroots movement, growing in tandem with online communities [1], has since found its way into even the medical mainstream, once notorious for pathologising autism. Leading psychiatric journals now advocate for interventions that work with autism, taking it as a point of departure rather than seeking to erase it [2, 3]. Autism funding, while remaining overwhelmingly directed at genetic and neurobiological research, has nonetheless seen a notable shift [4]. Driven in large part by the neurodiversity movement’s argument that autistic health ailments are not inextricable from autism itself, a growing share of resources is being channelled toward quality of life interventions across the lifespan. This is especially true of mental health, where an expanding body of work has begun to ask how autistic mental health problems can be addressed on their own terms, without assuming that autism is the underlying cause and without compromising neurodiversity-affirming principles in general [5–7].

What is common across most such approaches is a centring of autistic lived experience. This should be unsurprising, if only as a historical corrective. For decades, autistic people were given no voice in biomedical and mental health research, and interventions designed for them are now widely regarded as unethical, if not violating of human rights [8–10]. The awakening that the neurodiversity movement has brought about has crystallised into the idea that everyone but the population in question should take a step back and allow autistic people to define and describe what it is like to be autistic, and dictate what they need from the interventions that engage them. By the very terms in which it is articulated, this paradigm shift surfaces phenomenology, qua the philosophical method investigating the structure and meaning of experience, as its natural protagonist. A glance at a recent editorial from *The Lancet Psychiatry*, urging a recalibration of modern psychiatry more broadly, is instructive:

Implicit in any phenomenological project in psychiatry or psychology is a shift in patient state from object of study to subject whose perceptions and experiences are heard, accepted, and valued. This approach could redress power imbalances and generate collaborative projects in which all parties bring equally valued perspectives. Phenomenology cannot be the sole basis for mental health research. However, a greater number of dedicated investigations, and addition of a phenomenological dimension to larger projects, has the potential to advance – or at least unstick – multiple areas of mental health. There is no call to discard wholesale conceptual frameworks or the accumulated body of knowledge, but it is time to return to the things themselves [11].

While this general orientation would hardly find dissenters among contemporary neurodiversity scholars and advocates, a closer look at the literature reveals a rather complicated picture. Even among studies that revolve around autistic experience, actual phenomenological methods are a minority [12, 13]. Most research invoking lived experience relies on thematic analysis, opinion surveys, or loosely structured interviews, approaches far apart from phenomenological inquiry. When phenomenological methods are used, they tend to be justified on the broad idea that it is worthwhile to know what autistic experience is like across a variety of contexts. It is much rarer for researchers to ground their choice on phenomenology-specific and pragmatic terms: to answer why phenomenology, rather than any other method of perspective-taking, is best fitted to the topic at hand, and what, downstream, it concretely offers, e.g., as regards social or clinical intervention.

This paper tackles these two questions in the frame of what is probably the most urgent domain of research in autism studies: mental health interventions. Recent-decade research has documented that up to four in five autistic individuals meet criteria for at least one psychiatric disorder in their lifetime [14, 15], depression and anxiety being the most prevalent. Suicidality is a grave concern, with up to one in four autistic individuals attempting suicide in their lifetime and large cohort studies reporting a 2.56-fold increase in premature mortality [16–18]. Both autistic service users and practitioners have repeatedly placed autism-adapted interventions as a top priority [5, 19–21], yet the clinical reality lags far behind. Indicatively, a landmark UK NHS study of approximately 8,700 autistic adults found that autistic clients had lower odds of reliable recovery and higher odds of reliable deterioration than matched peers [22]. As such, if phenomenology can do uniquely necessary work in the mental health domain, as this paper argues, there is arguably no area of autism research where this is more important.

The paper is structured as follows. Phenomenology as a Distinctive Mode of Inquiry foregrounds three features that distinguish phenomenology from other ways of attending to experience. It also situates the paper among existing phenomenological accounts of autism and sets its stance: that autism, at the level of the condition, is not a deficit, while what serves a given individual remains, facet by facet, open to inquiry. The Verification Problem and the Pragmatist Criterion poses the problem of how one can tell that a phenomenological clarification has succeeded, and answers it with a pragmatist criterion of phenomenological success, drawn from the philosophy of science, on which an interpretation earns its credibility through what it lets us predict and do.

The rest of the paper outlines where phenomenology can do some of its most tangible clinical work in autism mental health: assessment and formulation, evaluation of outcomes, and the therapeutic relationship. The pragmatist verification criterion travels alongside analysis as the standard against which that work is judged. Before Treatment: Assessment and Formulation takes up where phenomenology can surface the experiential structure a presenting symptom obscures and bracket the habitual sorting of features into problem or difference. The Neurodiversity Tension, Two Safeguards, and a Circle, the methodological keystone, marks out the evaluation of outcomes as a second realm of phenomenological work, settling by the user’s lights what serves them facet by facet. It then defends the criterion against the circularity this invites and shows how phenomenological interpretation stays answerable to the autistic person it concerns. The Therapeutic Relationship (Coda) turns to the therapeutic relationship and reads it as an experiential phenomenon, turning the clinician’s attention reflexively onto their own (mis)attunement.

A few vignettes, drawn primarily from the NEUROPACT Pilot study [23], illustrate the analysis. The study was conducted by the author and colleagues and is, to our knowledge, the most granular investigation of autistic adults’ psychotherapy experiences to date. The patterns these cases illustrate, however, do not rest on this dataset alone, as each is triangulated against findings in the wider literature. Reliance on a single source for the illustrations reflects the current state of the field, in which experiential data of this granularity are, as yet, rarely collected.

The paper focuses throughout on the case of non-autistic clinicians working with autistic service users. The growing practice of autistic clinicians working with autistic service users raises its own phenomenological questions, but the available data on this configuration remain too sparse to support sustained analysis, and including it would complicate the argument without, at this stage, adding substance.

## Phenomenology as a Distinctive Mode of Inquiry

At least three things set phenomenology apart from other methods of paying heed to experience. The first is its structural and interpretive ambition. Phenomenology does not aim to capture the particular contents of one’s experience, but the invariant structures that persist across different kinds of experience and the meanings those structures carry. The structures can be thought of as the essential features without which a given type of experience would no longer be that type of experience [24, 25]. The meanings they carry are not the explicit beliefs or judgements a subject might report, but the significance experience already has by virtue of being organised the way it is. How phenomenologists reach these structures has varied: Husserl [24] sought to isolate them through systematic imaginative variation of experiential features; Heidegger [26] asked what must already be in place for everyday activity to be intelligible at all; and Merleau-Ponty [27] traced them through the body’s pre-reflective grip on the world. What unites these approaches is the ambition of identifying the skeletal architecture on which any particular experience is hung, and the significance it carries prior to any explicit act of reflection.

Two examples from mental health research illustrate the point. Ratcliffe [28] argues that depression is best understood not by cataloguing the specific thoughts or emotions that depressed persons report but by attending to a shift in existential feeling: the pre-intentional, embodied sense of belonging to a shared world that determines what kinds of intentional states one is capable of having at all. This identifies a transformation of the experiential field itself, which persists through the experience of any particular symptom. In autism research, Fuchs [29] likewise argues that autistic social differences are better explained not by reference to a faulty “theory of mind” module but by an altered intercorporeality: the pre-reflective, bodily attunement to others through which social understanding is ordinarily achieved [30, 31].

This latter account sits within a debate in which this paper must situate itself. Fuchs’ analysis, like much early phenomenological work on autism, has been read as proceeding from a whole-condition assumption of deficit, treating autism as a disturbance from the outset, and as such has drawn sustained criticism [13, 32, 33]. The analysis hereunder does not inherit this assumption. It is premised instead on the academically established and ethically weighty view of autism as value-neutral neurodiversity. Nonetheless, this is a commitment at the level of the condition, which does not settle the status of any particular facet of autistic experience, viz. whether a given feature is, for a given person in a given context, enabling or limiting. Boldsen’s [34, 35] work shows the two can even be held together. Re-reading the autistic orientation toward objects not as failed sociality but as a differently organised one, achieved through a shared relation to things rather than face-to-face attunement alone, Boldsen still speaks of a “shaky” attunement while denying that sociality is in any sense broken. How the bracketing this requires is sustained in clinical practice is taken up in The Neurodiversity Tension, Two Safeguards, and a Circle.

The second distinctive feature of phenomenology is its assumption that the invariant structures and meanings of experience are not transparent to the experiencing subjects. This contrasts sharply with approaches that treat first-person report as direct evidence of experiential reality. A person may be an authority on what they feel, want, or believe and yet poorly positioned to articulate the structural conditions that make those feelings, desires, and beliefs possible and meaningful – much as a fluent speaker is typically unable to state the grammatical rules that underpin their own speech [25]. Phenomenological clarification, then, is not a matter of listening more carefully to what people say about their experience, but of conducting conceptual and interpretive work to identify organising principles that typically exceed the subject’s self-understanding. In autism studies, this circumscription of epistemic authority raises an especially pointed complication, addressed in The Neurodiversity Tension, Two Safeguards, and a Circle.

Third, on most accounts, phenomenology demands a distinctive preparatory discipline of the inquirer: the suspension of what Husserl termed *the natural attitude*. The natural attitude is the default, taken-for-granted assumption that the world simply is as it presents itself to us, and that our categories for understanding it are unproblematic [24, 25]. This suspension, the *epoché*, does not involve doubting the existence of the world or denying the validity of scientific findings. It is, rather, a bracketing of one’s habitual theoretical commitments, so as to attend to how things show up in experience prior to the frameworks we ordinarily impose on them. The relevance for clinical contexts is immediate. A clinician approaching an autistic service user’s distress through the lens of, say, cognitive-behavioural theory will be predisposed to find dysfunctional cognitions and behaviours, while one operating psychodynamically will look for causal origins in early family relationships. The epoché asks the inquirer to hold such frameworks in abeyance – not to abandon them permanently, but to prevent them from doing the interpreting at first pass, so that the person’s experiential reality can come into view on its own terms, before it is sorted into the categories of any particular tradition [24, 36].

## The Verification Problem and the Pragmatist Criterion

If the structure and meaning of one’s experience are not transparent to them, as phenomenology wants, one might ask just what should count as evidence that a phenomenological interpretation has succeeded, i.e., that the structures and meanings arrived at are actual features of experience rather than artefacts of the method. Natural as this question may be, it has been rarely asked. From a disciplinary standpoint, this is unsurprising: phenomenology is a philosophical orientation, and philosophical orientations are not necessarily held to evidential standards. Nonetheless, the question is worth pressing, for two reasons. First, in at least most articulations, phenomenological inquiry purports not to impose structure and meaning upon experience by way, e.g., of a useful fiction, but to discover its features that are already there [25–27]. Therefore, a benchmark of this performance is a legitimate ask. Second, in psychology and psychiatry, the main foci of this paper and where phenomenology has gained the most traction, phenomenological findings are currently, or ideally, applied to aid clinical work [7, 28, 37, 38]. Assuming that the accuracy of these findings is a primary condition, if not a necessary one, for this task to succeed, we need some way of testing whether they are in fact accurate.

The criterion proposed here adapts a well-established move in the philosophy of science, namely, to judge a theory’s truth-likeness by what it achieves. The “no miracles” argument, the cornerstone of scientific realism, holds that the predictive and interventive success of mature science would be inexplicable, indeed a miracle, unless its theories were at least approximately true [39]. It would be a miracle, for instance, if electrons did not exist, given the success of electron theory in predicting cloud chamber phenomena and putting rockets to space. Experience is not mind-independent, and so the transposition to phenomenology cannot be wholesale. What carries over is the form of the “no miracles” inference, namely, that the predictive and interventive success of a phenomenological interpretation gives defeasible reason to think it has latched onto something real in the subject’s experiential organisation. Indeed, a genuine clarification of the structure of one’s experience ought to show downstream, e.g., in an enhanced capacity to anticipate what a situation will be like for them, and to design interventions that are meaningful and effective for them.

The proposal, then, is that phenomenological interpretations, in fields involving clinical application, should be judged by their capacity to facilitate clinical outcomes as valued by the service user. This last qualification is not incidental and, in the case of autism, it is not innocent. Why the measure of success must be the person’s own, rather than a standard clinical index, is a question that the neurodiversity commitments of this paper press hard in The Neurodiversity Tension, Two Safeguards, and a Circle.

Stated baldly, the criterion invites an objection, borne by the fact that clinical improvement has many possible authors. The therapeutic alliance is among the most robust predictors of outcomes across modalities; expectancy and placebo effects are pervasive; and much distress remits of its own accord, so that improvement may simply be regression to the mean [40–42]. Given this, how can improvement credit the interpretation in particular? The answer is that the criterion is not satisfied by improvement as such, but by improvement that follows the form of the analysis: change of the specific shape the interpretation predicts, in the place and along the lines it implies. Alliance, expectancy, and regression are, by their nature, nonspecific. They give no reason to expect improvement to track the particular content of one interpretation rather than another. Where it does so track, the best explanation is that the reading has identified something real. The point is sharpest when an intervention keyed to one interpretation succeeds where a competently delivered alternative, keyed to another, had failed.

A hypothetical will now demonstrate the shape of this inference, while Before Treatment: Assessment and Formulation will show it operating in real cases. Suppose that an autistic person seeking help for what has been diagnosed as social anxiety works with a phenomenologically trained therapist. Together, they uncover that the client’s avoidance of social functions does not stem from fear of negative evaluation, as the diagnostic label suggests, but from a disruption in the temporal structure of conversational exchange: a persistent lag between perceiving social cues and generating a response, such that by the time a response is formulated the moment has already passed, producing a chronic sense of being out of step. This approach can be deployed to intervene, say, by shifting therapeutic focus from cognitive restructuring of feared outcomes toward strategies that accommodate the temporal mismatch directly, e.g., slower-paced social formats, explicit pauses, and asynchronous communication. Should this succeed where the anxiety-focused treatment had not, the interpretation earns epistemic credit because it is hard to explain that differential success without granting that something about the client’s experience had been correctly identified.

A clarification guards this proposal against a likely misreading. To make clinical efficacy a measure of phenomenological success is neither to make phenomenology’s purpose clinical, nor to hold that phenomenological psychopathology should answer, as a field, to therapeutic outcome. Phenomenology in the mental health disciplines is largely descriptive and nosological, and its proximal aims are the discovery of structures of experience. What is proposed is only that one route to testing whether an interpretation has succeeded runs through what it lets us predict and do and that clinical efficacy is the form this route takes in the domain this paper concerns.

One such alternative route is subjective clarity, i.e., making the subject more aware of the meaning of their own experience, which some would hold to be an even more important measure of phenomenological success. This is especially so given that autistic people often report blocked access to that meaning, particularly regarding emotions [43], which is sometimes discussed under the heading of alexithymia, though this framing is not uncontested [44]. The appeal is obvious, but a feeling of clarity about one’s experience can be misleading [45, 46]. So, the phenomenologist faces the same question pushed back a step: how does one know that the experience of having clarified meaning corresponds to actual clarification, rather than confabulation? This problem is, too, taken up in The Neurodiversity Tension, Two Safeguards, and a Circle. For now, it suffices to note that the proposal leaves room for additional criteria while staking its primary claim on clinical efficacy.

With the pragmatist criterion in place, it is now time to turn to the first proposed area where phenomenology may do discernible clinical work in autism mental health care: assessment and formulation, where the structure beneath a presenting symptom is brought to light.

## Before Treatment: Assessment and Formulation

One of the most consistent findings of mental health studies with autistic adults is that an ordinary clinical picture may correspond to a very inordinary kind of underlying experience. Even more importantly, failing to notice this makes the difference between a successful and a failing intervention [10, 20, 21]. To illustrate, take the cases of two women from the NEUROPACT study. The first woman presented with what would be clinically described as an eating disorder. Her therapist responded with a standard protocol for the general population, trying to surface their client’s negative body representations and feelings of worth tied to a specific type of body. Of this, she said:

A lot of it didn’t really apply to me ... [Anorexia was approached through] questions like, how do you feel about your body image ... but a lot of my eating rules aren’t really related to body image stuff. I've now had my eating disorder diagnosis changed, which is a lot more relevant to me, because it’s a lot more about sensory issues … I didn’t really get [the initial approach], because I didn’t really have those … in the first place [23].

She then described working with a neurodiversity-informed clinician, where the angle shifted from body representations to sensory issues. Together, through examining her eating experiences, they unearthed that the woman had strong aversive responses to the texture of an array of foods, as well as mashing food in the mouth. Understanding this and finding workarounds – planning meals around specific sets of foods, serving them at different temperatures and textures, getting nutrients in liquid forms – was described by this woman as significantly improving her eating habits, which in turn helped her lead a healthier and happier lifestyle.

The second woman presented with acute distress in response to loud voices, serious enough to compromise her social life. Her psychodynamic therapist traced the response back to her mother shouting at her as a child and worked to bring that supposed early fear to conscious processing. It did not help:

On one hand, there’s the belief that I can’t stand loud noises because ... my mom shouted, and on the other, that I can’t stand loud noises because I’ve always had sensory issues. ... [S]ince I was young ... when I heard my mom shouting, I was locking myself in the bathroom, hitting myself and saying “Die”. But it was the sound that bothered me, not what she was saying. (ibid.)

The woman went on to describe working with other therapists, gradually shifting the explanatory focus from psychodynamic relations to sensory issues. She eventually settled on noise-cancelling devices, environmental adjustments, and directly negotiating her sensory needs with her peers. She outlined a much better outcome, concluding that she needed “headphones, not psychoanalysis” for the problem at hand.

These two cases are vivid, but the pattern they instantiate is not idiosyncratic to them. That a difficulty presenting as a psychiatric or behavioural problem can be sensory in its constitution is independently documented across autistic populations. What presents as anxiety often tracks sensory over-responsivity rather than feared outcomes [47, 48], and autistic eating difficulty is frequently driven by aversion to the sensory properties of food rather than body image [49, 50]. Just as well documented is that this constitution often goes missed. Autistic experience is subject to what is termed *diagnostic overshadowing*, whereby a presentation is overlooked or misattributed to a more familiar psychiatric category [51, 52]. In one recent large survey, one in four autistic adults reported having been misdiagnosed, with eating- and trauma-related formulations among the recurring substitutions [51].

Writing off such cases as therapist incompetence would be misguided. In the NEUROPACT study instances, both clinicians were well-credentialed, both followed standard, evidence-supported protocols, and nothing in the testimonies suggests inadequate demeanour or alliance-building. What went astray was more fundamental, viz. a failure to reckon with the fact that these clients’ experiences were underdetermined by their symptom presentation and that the structure and meaning of those experiences were, by way of the clients’ neurotype, likely different from the norm. In the first case, eating held no meaningful association with body image. What persisted across the woman’s encounters with food was a structure of sensory disgust. In the second, a structure of sensorily seated intolerability persisted across high-pitched sounds irrespective of their content, bearing no connection to early representations of the mother’s outbursts. In both, the presenting symptom was legible through the lens of the therapist’s training while the experience beneath it was not.

Places like these are where phenomenology earns its keep. In the cases of the two women, what eventually worked was an investigation attending to what each experience actually was like, its felt quality and invariance across content and context, until the sensory constitution of the difficulty became clear. The first woman did not walk into therapy knowing that her eating difficulties were constituted by sensory disgust; the second had provisionally accepted her therapist’s psychodynamic formulation but found it unfruitful. In both cases, interventions adjusted to the newly clarified structure succeeded. By the pragmatist criterion presented above, it is that success, judged from the women’s own perspective, which counts as evidence that the phenomenological work got something right, seeing as the improvement followed the form of the interpretation. The shape of the failure that preceded the success lends additional weight to these particular cases. The familiar nonspecific sources of clinical improvement – therapeutic alliance, expectancy, regression to the mean – cannot account for positive outcomes, since they are indifferent to the content of a formulation and so give no reason why help should have arrived only once the interpretation changed. That a competent prior formulation had failed, and the sensory one succeeded, is thus the sharpest available form of the predictive fit.

Admittedly, the therapists who got it right almost certainly did not employ phenomenology by name. There is no indicator for this in the data, and the use of formal phenomenological methods by practising clinicians remains relatively rare. However, what matters is that someone attended carefully to what it was like to be these clients in the moment of their distress, bracketed theoretical frameworks that had previously been imposed on that distress, in effect performing something very like the epoché, and arrived at an actionable understanding. The formal label, unlike the apparatus itself, is secondary. When clinical attentiveness of this kind succeeds without phenomenological training, it succeeds by talent or luck. Some clinicians simply notice what others miss, and there is no reliable way to transmit the skill across a profession. What phenomenology supplies is the conceptual scaffolding that makes such attentiveness trainable and answerable. The notion of invariant experiential structures gives clinicians something specific to attend to; the discipline of the epoché gives them a practicable method for suspending their own theoretical precommitments; and the pragmatist criterion gives them a standard against which to check their interpretations.

Formulation in autism makes a further demand of this discipline: that the clinician suspends not only their theoretical school but the reflexive treatment to sort an autistic feature as problem or difference before what it does for the person has been seen. How that openness is finally adjudicated is taken up in The Neurodiversity Tension, Two Safeguards, and a Circle.

Lastly for this section, a qualification about generalisability. Phenomenological investigation of this kind cannot be conducted with a representative sample (whatever that may mean in the case of autistic service users) and generalised from there. The fact that the anorexia of one or more service users is constituted by sensory disgust does not entail that every autistic person’s anorexia is constituted likewise. It would be implausible to suppose that autistic individuals are impervious to the causal factors operative in anorexia as it is typically understood. Phenomenological inquiry must be grounded in token cases, i.e., in the particular subjectivity and particular autism of each service user, rather than treated solely as a typological exercise yielding rules for general application. This, however, is not to say that phenomenological inquiry in autism is without general purchase. Compiled across enough cases, attention to what particular interventions are like from the inside can aggregate into rough directional rules – that forced masking is overwhelmingly experienced as harmful, that coerced eye contact procures no meaningful result [8, 9, 53] – which mark out interventions warranting particular caution and others that can generally be approached with more confidence.

## The Neurodiversity Tension, Two Safeguards, and a Circle

The pragmatist criterion and the formulation practice elaborated on above share a commitment that, in the autism context, is double-edged. Both go beneath what the service user can themselves articulate, to a structure that the non-transparency thesis holds to exceed their explicit grasp. This raises a pointed worry, flagged earlier. The neurodiversity movement was in large part a reclamation of epistemic authority; a demand that autistic people be believed about their own lives after a long history of being spoken for and explained away [33, 54]. A method that sets out to identify structures and meanings the subject cannot state, and to let those structures shape what counts as their good, can look like a return to precisely that displacement.

It is fundamentally important to notice that the worry of epistemic injustice must run in both directions. That is, treating a person’s surface report as the limit of what can be understood about their experience can itself fail them as knowers. It confines them to whatever vocabulary they already command and withholds the interpretive work that might let an inarticulate difficulty become sayable and so addressable. The first of the two NEUROPACT study women did not arrive able to say that her eating was organised by sensory disgust. An inquiry that had treated her self-report as the final word would have left her with the body image formulation that did not fit. Refusing to go deeper can erase the autistic voice as surely as overriding it can, only more quietly. The task is therefore not to choose between honouring self-report and going beyond it but to go beyond it in a way that remains answerable to the person. Two safeguards are put forward as securing that answerability.

The first is to return interpretations to the people they concern and to give their response genuine standing in deciding whether they hold. This practice is known in qualitative research as member-checking or respondent validation, widely employed in Interpretative Phenomenological Analysis [55] and kindred traditions. However, upon erecting this safeguard, one must reckon with two objections. The first is internal. If, as the above analysis of phenomenology as a distinctive mode of inquiry maintained, experiential structure is not transparent to the subject, why should their assent bear on whether an interpretation of that structure is correct? The answer lies in an asymmetry between recognition and articulation. The grammar analogy cuts both ways: a fluent speaker cannot state the rules governing their speech, yet reliably recognises that a given sentence sounds wrong. So too, a service user may be unable to articulate the structure of their experience and yet be well placed to recognise, when an interpretation is offered back, that it does or does not fit. Member-checking trades on recognition, not articulation, and recognition is just what the non-transparency thesis leaves the subject normally capable of.

The second objection is external. Member-checking is not, in fact, the standard route to validity in qualitative or phenomenological research, and in some strains, it is deliberately set aside. A wide repertoire of strategies establishes the fidelity of findings without consulting participants at all, for instance, internal coherence, comprehensiveness of description, fit with prior analyses, and corroboration among trained inquirers [56, 57]. The reply is that which strategy is apposite depends on what the inquiry is for. Levitt and colleagues [56] make validity goal-relative: integrity is a matter of fidelity to the phenomenon and utility for the aims of the study and what secures each varies with those aims. Descriptive or theoretical phenomenology, whose object is the invariant structure of a type of experience, answers chiefly to fidelity, and the structure of a type is not a single participant’s to ratify. The work at issue here is different: phenomenological interpretation is applied to a particular person’s clinical care, where the interpretation shapes what is done to and for them, and where they hold the most direct stake in its accuracy. In that applied case, returning the interpretation to the person is not an optional nicety but part of what makes it answerable to the one it concerns.

The second safeguard opens onto what is, in its own right, a further realm of phenomenological work: the evaluation of clinical outcomes. Autistic service users often report that the outcomes they value run a long way apart from those that standard clinical indices track. What they care about is less the reduction of autistic traits that a symptom scale would register, and more so being able to live without the exhaustion of constant masking, sustaining a few relationships that feel genuine, and a greater sense of autonomy and of being at home in their own life [58–60].

Assuming that success can indeed not be read off scales, the claim here is that phenomenology can provide an appropriate relevant register. The pragmatist criterion referred to “outcomes as valued by the service user”. It is now time to give that contention content. “The user’s own terms” does not refer to their verbal verdict, their satisfaction rating, or their agreement with the clinician’s rationale. These are reflective achievements, and the nontransparency thesis should make us wary of treating them as the final word. It means something more fundamental: the global, pre-reflective sense of how one finds oneself in the world – what Heidegger [26] called *Befindlichkeit*, attunement, and what Ratcliffe [28] analyses as existential feeling. This is the register in which one is, before any articulation, more or less at home in one’s life, more or less coping. To evaluate an outcome in the user’s own terms is to ask whether this global attunement has improved by their own lights; neither whether they can say why, nor whether their account of it matches the clinician’s.

The safeguard just outlined does its sharpest work where reflective verdicts come apart, and this is also where the deficit question is finally handled. The stance described earlier – nondeficit at the level of the condition, no verdict fixed in advance at the level of the facet – leaves open, for any given autistic feature, whether it is for a given individual enabling, limiting, or both. That openness is itself a discipline, a refusal to sort a facet into vice or virtue before what it does for the person has been seen. A monotropic attentional style is the plain case: for one person, or for the same person in a different setting, it can be the ground of deep expertise and absorbed flow, or a source of painful inflexibility [61]. What settles the issue is not a rule but the global register: whether, living with it one way or the other the person finds themselves more at home. The same holds when goods conflict outright. One NEUROPACT study participant described an intervention that had made him more socially legible to nonautistic others and thus less isolated but at the cost of a felt inauthenticity:

It did help, yes. Because I was acting more appropriately to the situations around me. But personally I wasn’t happy. I was confused about it because from the one side I was like, I’m being fake. I was not honest with them, but I was being appropriate with my reaction, so it was very confusing. … I would say that it was helpful. Because even though at those moments I wasn’t being entirely truthful, I was able to have people around me that I liked. I was happy to have people around me, going out with them, speaking to them, it allowed me to keep the connections more than the moments themselves [23].

No type-level rule resolves what this psychotherapy client is weighing. Connection and authenticity are both genuine goods, and which should yield is not a fact about autism but a fact about this life. On different days even, by the user’s own account, the balance can read differently. That this tension is not trivial is borne out at the population level: camouflaging of the kind he describes is associated with poorer mental health outcomes and, in several studies, with suicidality [62, 63]. What settles it is whether, living the one way or the other, this particular service user is on the whole more at home, enjoying more, suffering and being alienated less.

Taken together, the two safeguards expose the apparatus to a worrying circularity. Earlier, the pragmatist criterion was shown to verify a phenomenological interpretation by its clinical outcomes. Now, the second safeguard has made those outcomes themselves a matter of phenomenological assessment. So, an interpretation is vindicated by an outcome that is itself phenomenologically assessed, leaving the test looking like an expression of the very kind of thing it tests.

The circularity is actual, but not vicious. Seeing why turns on a distinction the objection runs together, between two levels of phenomenological assessment. A given phenomenological interpretation under test is granular: it concerns the specific structure of a particular difficulty, e.g., the sensory disgust organising eating or the intolerability beneath distress at sound. This is the level the nontransparency thesis governs. It exceeds the subject’s articulate grasp, which is why it needs verifying at all. By contrast, the outcome tested is global: it concerns Befindlichkeit, whether the person is, on the whole, more at home in their life. These are different objects of assessment, the crux being that the global one is not constituted by the granular one. A person can come to find themselves more at home, or fail to, independently of whether any particular interpretation of their difficulty was correct, because the global register answers to the whole texture of a life, of which the targeted difficulty is one strand. The testing level is thus not an echo of the tested level but an independent purchase on it. Independence, not certainty, is what a vicious circle lacks: a circle is vicious when the test merely restates what it tests, and this one does not.

This establishes what the argument needs, and only that: the global assessment is independent of the granular interpretation it checks. Importantly, it does not establish that the global assessment is reliable. Independence concerns whether the test presupposes its conclusion; reliability concerns how often the test is right. Indeed, a test may presuppose nothing and still, often enough, get the answer wrong. How far global self-assessment can be trusted, how Befindlichkeit is interrogated, how often it misreports, how such distortions are caught and corrected, all these are real and large questions left here as work still to be done.

With the circle met, the apparatus assembled across The Verification Problem and the Pragmatist Criterion, Before Treatment: Assessment and Formulation, The Neurodiversity Tension, Two Safeguards, and a Circle stands as an usable account of where phenomenology can be applied in clinical work with autistic service users and of what its success consists in and how it can be pinpointed. What remains is the therapeutic process that runs between assessment and outcome, to which a closing word is owed.

## The Therapeutic Relationship (Coda)

A full treatment of phenomenology’s bearing on the therapeutic relationship lies beyond this paper’s scope, but the apparatus already assembled extends to it cleanly, and the extension is worth marking. The therapeutic alliance is not merely a precondition of the work but an experiential phenomenon in its own right, inter alia in the felt quality of being understood, or failing to be, and it is among the most robust predictors of outcome across therapeutic modalities [40], including in therapy with autistic clients [64, 65]. In such therapy, that felt quality is shaped by what Milton [33] calls the double empathy problem, or the difficulty of mutual understanding between autistic and non-autistic people. A clinician who reads a client’s stillness, level affect, or averted gaze as disengagement or resistance may simply be registering the far side of that gap, construing an autistic way of being present through neurotypical expectations of what presence looks like.

This is where the epoché turns reflexively. In formulation, it was trained on the client’s experience; in the relational moment it must be trained also on the clinician’s own: on the interpretations they are placing, in real time, upon the client’s conduct, and on the sense of misattunement those interpretations produce. To bracket them is not to feign a blank neutrality but to hold one’s own reading open long enough to ask whether the gap in the room is the client’s to close. Often it is not, and recognising as much is itself part of building the alliance, since a client who senses that their way of being present is being read as failure has every reason to withdraw further. The discipline, in short, is the one the paper has described throughout, now exercised within the relation rather than upon a formulation – and answerable, in the end, to the same court: whether the relationship comes to feel, in the user’s own terms, like one in which they are met.

## Conclusion

This paper set out from two questions about phenomenology’s place in autism mental health: why it should be preferred to other ways of attending to experience, and what, tangibly, it is for. The answer to the first lies in what distinguishes phenomenology as a mode of inquiry: its ambition to reach the invariant structures beneath what a person reports, its assumption that those structures are not transparent to the subject who lives them, and the epoché by which the inquirer suspends the frameworks that would otherwise do the interpreting. The answer to the second question has two parts, and together they are the paper’s main contribution.

The first is a criterion of phenomenological success. Against the worry that phenomenological interpretation has no benchmark, it was proposed that an interpretation earns its credibility through what it lets us predict and do: its capacity, in the clinical case, to facilitate outcomes the service user values. The pragmatist criterion of success was defended against the charge that verifying interpretations through outcomes is circular by showing that the granular interpretation under test and the global register in which outcomes are felt are independent levels, so that the test is no echo of the thing it tests.

The second is a map of where this work is done. Phenomenology can do definite, non-substitutable work minimally at three points in autism mental health care: in assessment and formulation, where it surfaces the experiential structure a presenting symptom obscures; in evaluation of outcomes, where it supplies the very register in which success can be judged at all; and in the therapeutic relationship, where it turns the clinician’s attention reflexively onto their own attunement.

Two implications follow for the field. The first is practical: if phenomenological interpretations are to be verified by outcomes valued by the user, the means of eliciting and tracking such outcomes – instruments sensitive to the global register rather than to symptom counts alone – largely remain to be built. The second concerns the shape of phenomenological autism research itself. It should be both token- and type-level, grounded in the particular subjectivity of each service user while letting cautious directional rules accrue across cases; clinically embedded, since the criterion can only be applied where interpretations meet outcomes; and answerable, throughout, to the people it concerns.

## Conflict of Interest Statement

The author has no conflicts of interest to declare.

## Funding Sources

This research received no specific grant from any funding agency in the public, commercial, or not-for-profit sectors.

## Author Contributions

Themistoklis Pantazakos: conceptualization, investigation, methodology, writing – original draft, and writing – review and editing.

## References

[1] Silberman S . NeuroTribes: the legacy of autism and the future of neurodiversity. New York: Avery; 2015.

[2] Dawson G , Franz L, Brandsen S. At a crossroads–reconsidering the goals of autism early behavioral intervention from a neurodiversity perspective. JAMA Pediatr. 2022;176(9):839–40. 10.1001/jamapediatrics.2022.2299 PMC10069446 35816341

[3] Lord C , Charman T, Havdahl A, Carbone P, Anagnostou E, Boyd B, . The lancet commission on the future of care and clinical research in autism. Lancet. 2022;399(10321):271–334. 10.1016/S0140-6736(21)01541-5 34883054

[4] Interagency Autism Coordinating Committee . 2021–2023 IACC strategic plan for autism research, services, and policy. Washington (DC): U.S. Department of Health and Human Services; 2023. Available from: https://iacc.hhs.gov/publications/strategic-plan/2023/

[5] Camm-Crosbie L , Bradley L, Shaw R, Baron-Cohen S, Cassidy S. “People like me don’t get support”: autistic adults’ experiences of support and treatment for mental health difficulties, self-injury and suicidality. Autism. 2019;23(6):1431–41. 10.1177/1362361318816053 PMC6625034 30497279

[6] Pantazakos T . Neurodiversity and psychotherapy – connections and ways forward. Couns Psychother Res. 2025;25(1):e12675.

[7] Pantazakos T , Vanaken GJ. Addressing the autism mental health crisis: the potential of phenomenology in neurodiversity-affirming clinical practices. Front Psychol. 2023;14:1225152. 10.3389/fpsyg.2023.1225152 PMC10507173 37731874

[8] Dawson M . The misbehaviour of behaviourists: ethical challenges to the autism-ABA industry [Internet]. 2004. [cited 2025 August 13]. Available from: https://www.sentex.ca/∼nexus23/naa_aba.html

[9] Kupferstein H . Evidence of increased PTSD symptoms in autistic children and adolescents whose parents report exposing them to applied behaviour analysis. Adv Autism. 2018;4(1):19–29.

[10] Pantazakos T . Treatment for whom? Towards a phenomenological resolution of controversy within autism treatment. Stud Hist Philos Biol Biomed Sci. 2019;77:101176. 10.1016/j.shpsc.2019.04.001 31003862

[11] The Lancet Psychiatry . The things themselves [editorial]. Lancet Psychiatry. 2021;8(3):169. 10.1016/S2215-0366(21)00033-X 33610216

[12] Howard K , Katsos N, Gibson J. Using interpretative phenomenological analysis in autism research. Autism. 2019;23(7):1871–6. 10.1177/1362361318823902 30672307

[13] Hughes E , Ekdahl D, Boldsen S. Between essence and diversity: challenges for phenomenological autism research. Theor Psychol. 2025;35(3):293–314.

[14] Hossain MM , Khan N, Sultana A, Ma P, McKyer ELJ, Ahmed HU, . Prevalence of comorbid psychiatric disorders among people with autism spectrum disorder: an umbrella review of systematic reviews and meta-analyses. Psychiatry Res. 2020;287:112922. 10.1016/j.psychres.2020.112922 32203749

[15] Lever AG , Geurts HM. Psychiatric co-occurring symptoms and disorders in young, middle-aged, and older adults with autism spectrum disorder. J Autism Dev Disord. 2016;46(6):1916–30. 10.1007/s10803-016-2722-8 PMC4860203 26861713

[16] Cassidy S , Bradley P, Robinson J, Allison C, McHugh M, Baron-Cohen S. Suicidal ideation and suicide plans or attempts in adults with Asperger’s syndrome attending a specialist diagnostic clinic: a clinical cohort study. Lancet Psychiatry. 2014;1(2):142–7. 10.1016/S2215-0366(14)70248-2 26360578

[17] Hirvikoski T , Mittendorfer-Rutz E, Boman M, Larsson H, Lichtenstein P, Bölte S. Premature mortality in autism spectrum disorder. Br J Psychiatry. 2016;208(3):232–8. 10.1192/bjp.bp.114.160192 26541693

[18] Newell V , Phillips L, Jones C, Townsend E, Richards C, Cassidy S. A systematic review and meta-analysis of suicidality in autistic and possibly autistic people without co-occurring intellectual disability. Mol Autism. 2023;14(1):12. 10.1186/s13229-023-00544-7 PMC10018918 36922899

[19] Maddox BB , Crabbe S, Beidas RS, Brookman-Frazee L, Cannuscio CC, Miller JS, . “I wouldn’t know where to start”: perspectives from Clinicians, agency leaders, and autistic adults on improving community mental health services for autistic adults. Autism. 2020;24(4):919–30. 10.1177/1362361319882227 PMC7192780 31674198

[20] Miller PK , Bowden SLJ, Dewison N, Ingham B, Thwaites R, Dagnan D. “Sometimes i’m feeling baffled and they’re probably feeling baffled”: on the experiences of psychological therapists working with autistic people in a structured primary care service for anxiety disorders and depression. Autism. 2025;29(10):2477–88. 10.1177/13623613251341610 PMC12417598 40401511

[21] Petty S , Bergenheim ML, Mahoney G, Chamberlain L. Adapting services for autism: recommendations from a specialist multidisciplinary perspective using freelisting. Curr Psychol. 2021;42(9):7489–500.

[22] El Baou C , Bell G, Saunders R, Buckman JEJ, Mandy W, Dagnan D, . Effectiveness of primary care psychological therapy services for treating depression and anxiety in autistic adults in England: a retrospective, matched, observational cohort study of national health-care records. Lancet Psychiatry. 2023;10(12):944–54. 10.1016/S2215-0366(23)00291-2 37977697

[23] Pantazakos T , Phua M, Vanaken GJ. “I really acted like I had no body; I was all mind”–neurodiversity-affirming psychotherapy for autistic adults: focus on concrete adaptations and lived experience shifts. Forthcoming; 2026.

[24] Husserl E . Ideas pertaining to a pure phenomenology and to a phenomenological philosophy, first book: general introduction to a pure phenomenology. Kersten F, translator. The Hague: Martinus Nijhoff; 1982. (original work published 1913).

[25] Zahavi D . Husserl’s phenomenology. Stanford (CA): Stanford University Press; 2003.

[26] Heidegger M . Being and time. Macquarrie J, Robinson E, translators. New York: Harper & Row; 1962. (original work published 1927).

[27] Merleau-Ponty M . Phenomenology of perception. Smith C, translator. London: Routledge & Kegan Paul; 1962. (original work published 1945).

[28] Ratcliffe M . Experiences of depression: a study in phenomenology. Oxford: Oxford University Press; 2015.

[29] Fuchs T . Pathologies of intersubjectivity in autism and schizophrenia. J Conscious Stud. 2015;22(1–2):191–214. https://www.ingentaconnect.com/content/imp/jcs/2015/00000022/F0020001/art00017

[30] De Jaegher H . Embodiment and sense-making in autism. Front Integr Neurosci. 2013;7:15. 10.3389/fnint.2013.00015 PMC3607806 23532205

[31] Gallagher S . Understanding interpersonal problems in autism: interaction theory as an alternative to theory of mind. Philos Psychiatr Psychol. 2004;11(3):199–217.

[32] Bottema-Beutel K , Kapp SK, Lester JN, Sasson NJ, Hand BN. Avoiding ableist language: suggestions for autism researchers. Autism Adulthood. 2021;3(1):18–29. 10.1089/aut.2020.0014 PMC8992888 36601265

[33] Milton DEM . On the ontological status of autism: the ‘double empathy problem’. Disabil Soc. 2012;27(6):883–7.

[34] Boldsen S . Social interaction style in autism: an inquiry into phenomenological methodology. J Phenomenol Psychol. 2021;52(2):157–92.

[35] Boldsen S . Material encounters: a phenomenological account of social interaction in autism. Philos Psychiatr Psychol. 2022;29(3):191–208.

[36] Husserl E . The crisis of European sciences and transcendental phenomenology: an introduction to phenomenological philosophy. Carr D, translator. Evanston (IL): Northwestern University Press; 1970. (original work published 1936).

[37] Parnas J , Møller P, Kircher T, Thalbitzer J, Jansson L, Handest P, . EASE: examination of anomalous self-experience. Psychopathology. 2005;38(5):236–58. 10.1159/000088441 16179811

[38] Pantazakos T . Scientific realism and phenomenology through the case study of autism [doctoral dissertation]. London: University College London; 2019. Available from: https://discovery.ucl.ac.uk/id/eprint/10082172/

[39] Psillos S . Scientific realism: how science tracks truth. London: Routledge; 1999.

[40] Flückiger C , Del Re AC, Wampold BE, Horvath AO. The alliance in adult psychotherapy: a meta-analytic synthesis. Psychotherapy. 2018;55(4):316–40. 10.1037/pst0000172 29792475

[41] Hengartner MP . Is there a genuine placebo effect in acute depression treatments? A reassessment of regression to the mean and spontaneous remission. BMJ Evid Based Med. 2020;25(2):46–8. 10.1136/bmjebm-2019-111161 30975717

[42] Whiteford HA , Harris MG, McKeon G, Baxter A, Pennell C, Barendregt JJ, . Estimating remission from untreated major depression: a systematic review and meta-analysis. Psychol Med. 2013;43(8):1569–85. 10.1017/S0033291712001717 22883473

[43] Kinnaird E , Stewart C, Tchanturia K. Investigating alexithymia in autism: a systematic review and meta-analysis. Eur Psychiatry. 2019;55:80–9. 10.1016/j.eurpsy.2018.09.004 PMC6331035 30399531

[44] Williams ZJ , Gotham KO. Improving the measurement of alexithymia in autistic adults: a psychometric investigation of the 20-item Toronto alexithymia scale and generation of a general alexithymia factor score using item response theory. Mol Autism. 2021;12(1):56. 10.1186/s13229-021-00463-5 PMC8353782 34376227

[45] Nisbett RE , Wilson TD. Telling more than we can know: verbal reports on mental processes. Psychol Rev. 1977;84(3):231–59.

[46] Schwitzgebel E . The unreliability of naive introspection. Philos Rev. 2008;117(2):245–73.

[47] Green SA , Hernandez L, Tottenham N, Krasileva K, Bookheimer SY, Dapretto M. Neurobiology of sensory overresponsivity in youth with autism spectrum disorders. JAMA Psychiatry. 2015;72(8):778–86. 10.1001/jamapsychiatry.2015.0737 PMC4861140 26061819

[48] Green SA , Ben-Sasson A. Anxiety disorders and sensory over-responsivity in children with autism spectrum disorders: is there a causal relationship? J Autism Dev Disord. 2010;40(12):1495–504. 10.1007/s10803-010-1007-x PMC2980623 20383658

[49] Bourne L , Mandy W, Bryant-Waugh R. Avoidant/restrictive food intake disorder and severe food selectivity in children and young people with autism: a scoping review. Dev Med Child Neurol. 2022;64(6):691–700. 10.1111/dmcn.15139 35112345

[50] Nimbley E , Golds L, Sharpe H, Gillespie-Smith K, Duffy F. Sensory processing and eating behaviours in autism: a systematic review. Eur Eat Disord Rev. 2022;30(5):538–59. 10.1002/erv.2920 PMC9545673 35737818

[51] Kentrou V , Livingston LA, Grove R, Hoekstra RA, Begeer S. Perceived misdiagnosis of psychiatric conditions in autistic adults. eClinicalMedicine. 2024;71:102586. 10.1016/j.eclinm.2024.102586 PMC11001629 38596613

[52] Van Schalkwyk GI , Peluso F, Qayyum Z, McPartland JC, Volkmar FR. Varieties of misdiagnosis in ASD: an illustrative case series. J Autism Dev Disord. 2015;45(4):911–8. 10.1007/s10803-014-2239-y 25218849

[53] Kapp SK , Steward R, Crane L, Elliott D, Elphick C, Pellicano E, . “People should be allowed to do what they like”: autistic adults’ views and experiences of stimming. Autism. 2019;23(7):1782–92. 10.1177/1362361319829628 PMC6728747 30818970

[54] Fricker M . Epistemic injustice: power and the ethics of knowing. Oxford: Oxford University Press; 2007.

[55] Smith JA , Flowers P, Larkin M. Interpretative phenomenological analysis: theory, method and research. London: Sage; 2009.

[56] Levitt HM , Motulsky SL, Wertz FJ, Morrow SL, Ponterotto JG. Recommendations for designing and reviewing qualitative research in psychology: promoting methodological integrity. Qual Psychol. 2017;4(1):2–22.

[57] Stanghellini G . The grammar of the psychiatric interview: a plea for the second-person mode of understanding. Psychopathology. 2007;40(2):69–74. 10.1159/000098486 17215592

[58] McConachie H , Mason D, Parr JR, Garland D, Wilson C, Rodgers J. Enhancing the validity of a quality of life measure for autistic people. J Autism Dev Disord. 2018;48(5):1596–611. 10.1007/s10803-017-3402-z PMC5889785 29188584

[59] Nicolaidis C , Raymaker DM, McDonald KE, Lund EM, Leotti S, Kapp SK, . Creating accessible survey instruments for use with autistic adults and people with intellectual disability: lessons learned and recommendations. Autism Adulthood. 2020;2(1):61–76. 10.1089/aut.2019.0074 PMC7188318 32355908

[60] Pellicano E , Heyworth M. The foundations of autistic flourishing. Curr Psychiatry Rep. 2023;25(9):419–27. 10.1007/s11920-023-01441-9 PMC10506917 37552401

[61] Murray D , Lesser M, Lawson W. Attention, monotropism and the diagnostic criteria for autism. Autism. 2005;9(2):139–56. 10.1177/1362361305051398 15857859

[62] Cassidy SA , Gould K, Townsend E, Pelton M, Robertson AE, Rodgers J. Is camouflaging autistic traits associated with suicidal thoughts and behaviours? Expanding the interpersonal psychological theory of suicide in an undergraduate student sample. J Autism Dev Disord. 2020;50(10):3638–48. 10.1007/s10803-019-04323-3 PMC7502035 31820344

[63] Khudiakova V , Russell E, Sowden-Carvalho S, Surtees ADR. A systematic review and meta-analysis of mental health outcomes associated with camouflaging in autistic people. Res Autism Spectr Disord. 2024;118:102492.

[64] Albaum CS , Vashi N, Bohr Y, Weiss JA. A systematic review of therapeutic process factors in mental health treatment for autistic youth. Clin Child Fam Psychol Rev. 2023;26(1):212–41. 10.1007/s10567-022-00409-0 PMC9879813 35999330

[65] Brewe AM , Mazefsky CA, White SW. Therapeutic alliance formation for adolescents and young adults with autism: relation to treatment outcomes and client characteristics. J Autism Dev Disord. 2021;51(5):1446–57. 10.1007/s10803-020-04623-z PMC7856227 32737670

